# The Identification of Biomarkers and Therapeutic Targets for Diabetic Kidney Disease by Integrating the Proteome with the Genome

**DOI:** 10.3390/biomedicines13040971

**Published:** 2025-04-16

**Authors:** Yuefeng Yu, Jiang Li, Bowei Yu, Yuetian Yu, Ying Sun, Yuying Wang, Bin Wang, Kun Zhang, Mengjun Tang, Yingli Lu, Ningjian Wang

**Affiliations:** 1 Institute and Department of Endocrinology and Metabolism, Shanghai Ninth People’s Hospital, Shanghai Jiao Tong University School of Medicine, Shanghai 200011, China; yuyuefeng981125867@163.com (Y.Y.); a1670035237@163.com (J.L.);; 2 The 967th Hospital of Joint Logistic Support Force of People’s Liberation Army, Dalian 116011, China; tmjchina@126.com

**Keywords:** diabetic kidney disease, protein, mendelian randomization, drug target

## Abstract

**Background:** The blood proteome is a major source of biomarkers and therapeutic targets. We conducted a proteome-wide Mendelian randomization (MR) study to identify cardiometabolic protein markers for diabetic kidney disease (DKD). **Methods:** We measured all 369 proteins in the Olink Explore 384 Cardiometabolic and Cardiometabolic panel of 500 patients with type 2 diabetes from 11 communities in Shanghai. Protein quantitative trait loci (pQTLs) were derived by coupling genomic and proteomic data. Cis-pQTLs identified for proteins were used as instrumental variables in MR analyses of DKD risk, and the outcome data were obtained from 8401 Japanese individuals with type 2 diabetes (2809 cases and 5592 controls). Replication MR analysis was performed in the UK Biobank Pharma Proteomics Project (UKB-PPP). Colocalization analysis and the Heidi test were used to examine whether the identified proteins and DKD shared causal variants. **Results:** Among the 369 proteins, we identified 66 independent cis-pQTLs for 64 proteins. MR analysis suggested that two cardiometabolic proteins (UMOD and SIRPA) may play a causal role in increasing DKD risk, with UMOD showing replication in UKB-PPP. Bayesian colocalization further supported the causal effects of these proteins. Additional analyses indicated that UMOD is highly expressed in renal macrophages. Further downstream analyses suggested that UMOD could be a potential novel target and that SIRPA could be a potential repurposing target for DKD; however, further validation is needed. **Conclusions:** By integrating proteomic and genetic data from patients with type 2 diabetes, we identified two protein biomarkers potentially associated with DKD risk. These findings provide insights into DKD pathophysiology and therapeutic target development, but further replication and functional studies are needed to confirm these associations.

## 1. Introduction

Diabetic kidney disease (DKD) is a prominent complication of diabetes mellitus, affecting approximately 50% of patients with DM globally [1]. DKD eventually progresses to end-stage renal disease (ESRD) in the absence of aggressive interventions [2]. Despite the increasing global burden of DKD, treatment options remain limited, with most therapies involving antidiabetic drugs that only slow disease progression rather than providing a cure [3,4]. Furthermore, some antidiabetic drugs are contraindicated in advanced DKD patients, whereas others require dose adjustments due to a heightened risk of drug toxicity [5]. As the prevalence of diabetes continues to rise, the healthcare burden associated with DKD is expected to escalate, emphasizing the urgent need for a deeper understanding of its pathogenesis and the development of targeted therapeutic interventions.

Metabolic dysregulation, including hyperglycemia, hyperlipidemia, and insulin resistance, acts as the initiating step of DKD and has a long-term programming effect on DKD progression [3,6]. Nevertheless, the identification of proteins that reflect these metabolic changes and contribute to DKD remains limited. As the main effector molecules of cells and biological processes and the ultimate products of gene expression, proteins can act as major sources of biomarkers and targets of most drugs [7,8]. Proteomics-based biomarker discovery has emerged as a powerful strategy for identifying disease-related proteins that could serve as potential therapeutic targets. Given that most FDA-approved drugs target proteins [9], systematic proteome-wide screening offers a unique opportunity to identify causal biomarkers and druggable targets for diseases such as DKD. However, distinguishing causative from correlational protein–disease associations remains a major challenge, as protein levels can be influenced by various confounding factors, such as disease progression, treatment effects, and environmental influences.

To address these challenges, Mendelian randomization (MR) leverages genetic variants associated with protein levels—in the form of protein quantitative trait loci (pQTLs)—as instrumental variables to infer causal relationships between circulating proteins and disease risk. MR design mimics a natural randomized trial to elucidate causal relationships between exposure and disease, minimizing reverse causation and confounding bias [10]. Recent proteome-wide MR studies have demonstrated the utility of MR in uncovering causal protein–disease links and prioritizing druggable proteins. However, a single MR finding does not guarantee robust protein–disease associations. To strengthen causal inference, colocalization analysis is usually conducted to examine whether the genetic signals for protein levels and disease risk originate from the same underlying causal variant rather than from linkage disequilibrium. Understanding the cellular context of proteins using single-cell RNA sequencing helps identify which cell types are most affected by the disease. Additionally, assessing druggability through databases such as Open Targets and DrugBank enables the prioritization of proteins with existing therapeutics, facilitating drug repurposing and translational applications [11,12]. However, to date, no proteome-wide MR study focusing on DKD has been conducted using the Olink platform in a Chinese population, leaving a critical gap in understanding DKD-related proteomics.

In this study, we aimed to identify circulating cardiometabolic proteins associated with DKD by integrating plasma proteomic and genomic data through proteome-wide MR analysis followed by colocalization analysis. The results of this study, which utilizes a Chinese population-based proteomic dataset, provide novel insights into potential causal protein biomarkers and therapeutic targets for DKD, addressing a critical gap in the understanding of the molecular mechanisms underlying its development.

## 2. Methods

### 2.1. Study Population

The METAL study (Environmental Pollutant Exposure and Metabolic Diseases in Shanghai, http://www.chictr.org.cn (accessed on 13 April 2025), ChiCTR1800017573) involved an approximately 5000-person cohort of individuals with type 2 diabetes from 11 communities in Shanghai, China, within which 957 people completed the 5-year follow-up in 2023. Details of the study design and methods have been previously described [9,13]. In the present study, we randomly selected 500 patients from those who completed the 5-year follow-up to complete plasma protein measurements. Among the 500 patients, a total of 99 patients had DKD. This study was approved by the Ethics Committee of Shanghai Ninth People’s Hospital, Shanghai Jiao Tong University School of Medicine (Approval No. SH9H-2023-T142-1), and all participants provided written informed consent.

### 2.2. Proteomic Profiling

Baseline serum samples were collected by venipuncture in the morning after an overnight fast of at least 8 h and were refrigerated immediately. The refrigerated blood was centrifuged within 2 h after phlebotomy, and the serum was aliquoted and stored in a −80 °C freezer. Plasma proteomics was performed using the Olink Explore 384 Cardiometabolic panel (Olink Proteomics AB, Uppsala, Sweden), which combines the proximity extension assay (PEA) technique with next-generation sequencing (NGS). Specifics regarding the assay have been described in detail previously [14]. In brief, PEA technology uses two separate unique antibody probes, each of which is labeled with complementary single-stranded oligonucleotides. The probes bind to target antigens, producing a binding complex where the complimentary oligonucleotides exist close to each other, enabling the formation of a target sequence that can be quantified via next-generation sequencing (NGS) [15]. The sequence data were processed and normalized to generate normalized protein expression (NPX) values using Olink’s relative protein quantification unit on a log2 scale.

Stringent quality control was performed during the whole process to eliminate systemic and technical variance, and only samples and proteins that underwent the quality control process, including quality control (QC) and assay warnings, were used in this study. Specifically, the sample was flagged for a quality control (QC) warning if the average matched count (the number of reads for each specific combination of the sample and assay) was <500. The assay received a QC warning if the deviation in the median of the negative controls was >5 standard deviations from the set predefined value. In addition, the limit of detection (LOD) was determined using negative control samples. A principal component analysis of entire proteomic profiles was conducted to detect extreme outliers. Among the 500 patients, 491 passed quality control protein measurement. Among the 369 proteins measured, 365 passed quality control, and 359 (98.36%) were detected in >50% of the samples when their NPX was above the LOD (Supplementary Table S1). The ranges per protein (calculated as the 90th percentile—the 10th percentile) varied between 0.56 NPX and 9.96 NPX, with an average of 1.57 NPX (Supplementary Figure S1). No obvious outliers were observed in the principal component analysis (Supplementary Figure S2).

### 2.3. Genotyping

Genome-wide genotyping of the METAL study samples (*n* = 957) was performed via the SNP array, and sample and variant quality control was performed according to previous protocols. Samples were excluded on the basis of a call rate < 98%, PI_HAT value > 0.185, heterozygosity outliers, and sex discordance checks. Variants were excluded if the call rate was <98% or if they had a Hardy–Weinberg Equilibrium *p* < 1 × 10^−6^. Genotype imputation was performed using the 1000G Phase3 GRCh37 as a reference. Variants with imputation quality INFO < 0.5 or MAF < 0.01 were excluded. The final dataset included 4,874,824 variants.

### 2.4. Protein QTL Mapping

The pQTL discovery analysis was performed using an additive model after adjusting for age, sex, communities, and 10 genetic principal components in PLINK 2.0. Cis-SNPs within a 1 Mb window from the protein-coding gene were tested for their associations with the respective circulating protein levels. We filtered those variants that met stringent genome-wide (*p* < 5 × 10^−8^) significance in linear regression models and clumped them on the basis of the 1000 Genomes East Asian panel (R^2^ < 0.001).

### 2.5. MR Analysis

We performed two-sample MR analyses using the proteins with cis-pQTLs identified as exposure and GWASs of DKD as outcomes. Independent cis-pQTL variants identified in the present study were used as instrumental variables. The GWAS summary statistics for DKD were from 8401 Japanese individuals with type 2 diabetes (2809 cases and 5592 controls) [16]. Protein-associated SNPs that were not available in the outcome data were substituted with SNP proxies that exhibited a high-linkage disequilibrium (R^2^ ≥ 0.8) on the basis of the 1000 Genomes East Asian panel [11]. Missing SNPs without suitable SNP proxies were removed from the analysis. The *F* statistic was calculated to estimate the strength of the genetic instruments. In the case of a single independent instrumental variable, the Wald ratio was applied; otherwise, inverse-variance weighted (IVW) estimates were reported. To account for multiple testing, *p* < 0.05 after the Benjamini–Hochberg false discovery rate (FDR) adjustment was considered statistically significant. MR analyses were performed using the two-sample MR and Mendelian randomization packages in R software (4.2.2).

### 2.6. Replication MR Analysis

In the replication MR analysis, we further conducted two-sample MR analyses of the proteins identified in METAL using cis-PQTLs obtained from the UK Biobank Pharma Proteomics Project (UKB-PPP), which conducted proteomic profiling on blood plasma samples from 54,219 participants using the Olink platform [17]. The summary data of the genetic associations for DKD were obtained from 5717 European subjects (3345 cases and 2372 controls) [18].

### 2.7. Colocalization Analysis and Heterogeneity in Dependent Instrument (HEIDI) Analysis

To test whether the identified associations of proteins with DKD were driven by a shared causal variant or linkage disequilibrium, we conducted Bayesian colocalization analysis via the “coloc” package. The analysis was based on a Bayesian model that assesses support for five exclusive hypotheses: (1) no causal variant for either trait (H0); (2) causal variant for trait 1 only (H1); (3) causal variant for trait 2 only (H2); (4) two distinct causal variants for two traits (H3); and (5) a shared causal variant for both traits (H4) [19]. If the posterior probability for H4 (PP4) was greater than 0.8, two signals were considered to have strong evidence of colocalization. We also conducted a HEIDI test to further determine whether there was a single causal variant underlying the association between a protein and a disease. A *p*-value > 0.05 distinguished associations caused by the same SNP from associations caused by two SNPs with linkage disequilibrium.

### 2.8. Differential Gene Expression Analysis

The cell type-specific expression of target genes was further evaluated using single-cell RNA-seq data of human kidney tissue from the Tabula Sapiens Consortium [20], which could provide evidence of a potential causal effect on DKD at the plasma protein level. The differential gene expression between each cell type and other cell types was determined via Wilcoxon’s rank sum test to examine whether identified DKD causal protein-coding genes were highly expressed in a particular cell type in the kidney.

### 2.9. Druggability Analysis and Protein–Protein Interaction (PPI)

To assess the potential of the identified proteins as therapeutic targets, we searched the Drug–Gene Interaction Database [21], DrugBank [22], and the Open Targets [23] platform. In addition, we investigated the targets of known medications for the treatment of DKD using the Open Targets database. A protein–protein interaction (PPI) network was constructed to explore the potential interactions between the identified targets and drug targets for DKD using the STRING database [24].

## 3. Results

**Figure 1:**
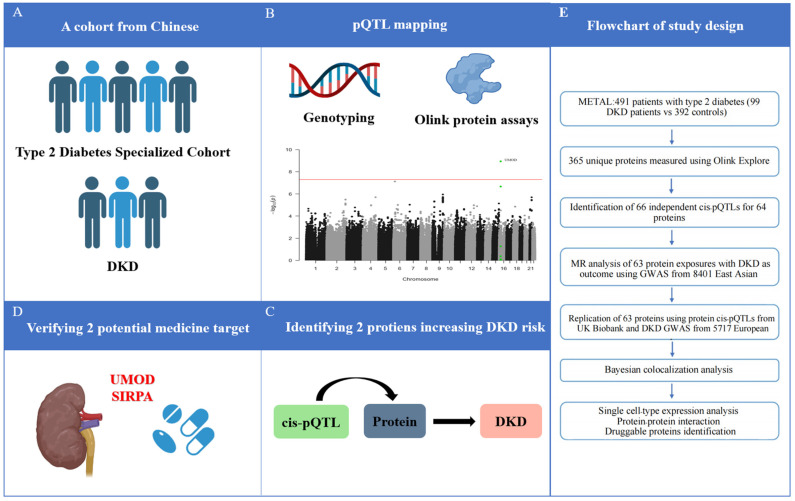
Schematic of the study. (**A**) Cohort selection; (**B**) pQTL mapping; (**C**) Identifying DKD-related circulating proteins; (**D**) verifying potential medicine target; (**E**) Flowchart of study design. DKD, diabetic kidney disease; GWAS, genome-wide association studies; MR, Mendelian randomization; pQTL, protein quantitative trait loci.

Overall, patients with DKD were more likely to be men, to be less educated, and to be current smokers and drinkers. DKD patients also had a higher BMI and a higher incidence of hypertension and dyslipidemia (Table 1). Figure 1 provides an overview of the study design.

### 3.1. Identification of Cis-pQTLs

The biological signals detected in the current study are shown using UMOD as an example (Supplementary Figure S3). After testing the association of genetic variants within a range of 1 Mb of genes encoding each of the 365 unique proteins with the levels of the corresponding proteins, a total of 5057 variants showed significant associations with 64 proteins, which were, thus, referred to as cis-pQTLs (Supplementary Table S2). After these genetic variants were clumped to a linkage disequilibrium (LD) of R^2^  <  0.001, 66 independent cis-pQTLs were identified, and the instrument strength (F statistics > 30) was met (Supplementary Table S3). An inverse relationship was observed between the effect size and effect allele frequency (EAF), with rarer pQTL variants generally exhibiting larger effect sizes (Supplementary Figure S4).

### 3.2. Identification of DKD-Related Circulating Cardiometabolic Proteins

**Figure 2:**
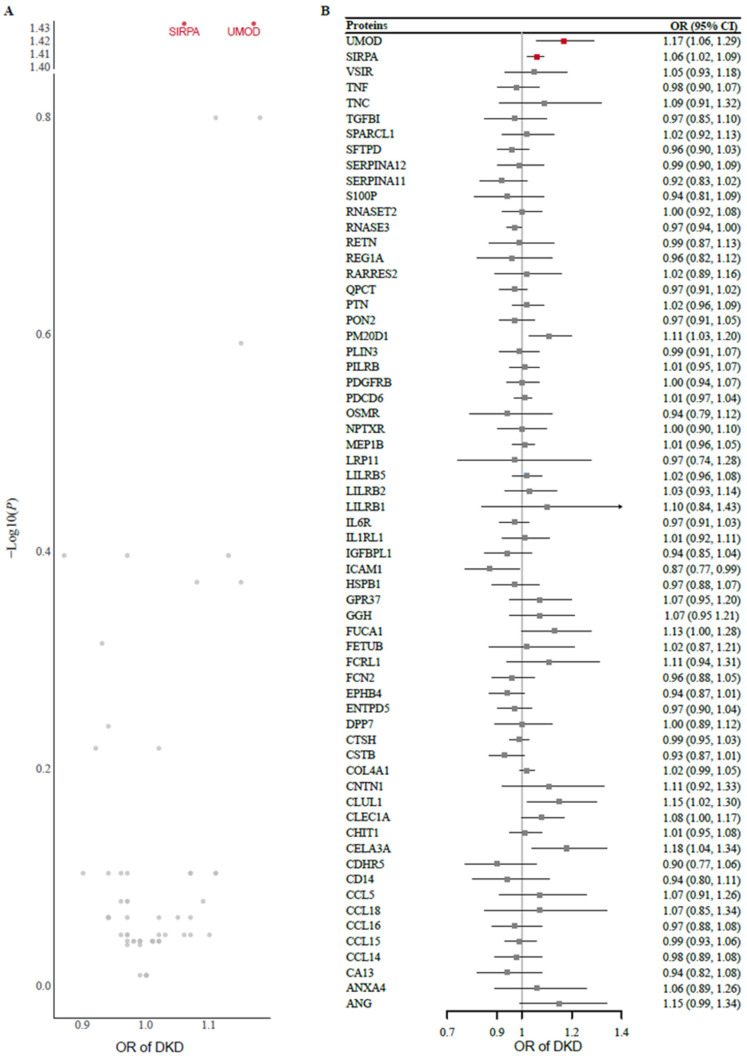
(**A**) Volcano plot and (**B**) forest plot of the MR analysis of 63 proteins and the risk of DKD. Odds ratios (ORs) for the increased risk of DKD were expressed as the per unit increase in protein levels. DKD, diabetic kidney disease; MR, Mendelian randomization.

Two-sample MR analysis using protein exposure from the cis-pQTL in the METAL study was conducted to investigate potential causal effects on DKD risk using outcome data from 8401 Japanese individuals with type 2 diabetes. After one protein (MSMB) without genetic instruments or SNP proxies was removed from the outcome data, 63 protein exposures were tested. The *F* statistics of all the genetic instruments were greater than 30, indicating good strength (Supplementary Table S3). Using the Wald ratio or IVW method, we found that genetically predicted higher levels of UMOD (odds ratio [OR] 1.17 [95% CI 1.06, 1.29]) and SIRPA (1.06 [1.02, 1.09]) were significantly associated with an increased risk of DKD after FDR correction for multiple testing (Figure 2). A full summary of the MR results is provided in Supplementary Table S4. In the replication analysis of the European dataset, we observed potential evidence of replication for UMOD (1.17 [1.03, 1.34]) but not for SIRPA (1.08 [0.98, 1.18]).

To explore the tissue-specific transcriptional effects of the identified proteins on DKD risk, we performed SMR using eQTL data from 50 tissues provided by the GTEx consortium (Supplementary Figure S5A). For *UMOD*, eQTLs were identified in only five tissues, and a significant causal association with DKD risk was found in pancreatic tissue. Although eQTLs for *UMOD* were detected in kidney tissue, no overlapping instrumental variants were identified when matched with DKD GWAS data, preventing MR estimation. *SIRPA* had eQTLs identified across all 50 tissues, and significant associations with DKD risk were observed for its RNA expression in the kidney, pancreas, small intestine, brain amygdala, and vaginal tissues.

We further investigated causal associations between the identified proteins and DKD-related phenotypes (Supplementary Figure S5B). *UMOD* showed consistent positive associations with eGFR, UACR, serum cystatin C level, serum creatinine level, and B2M level. *SIRPA* demonstrated potential causal associations with eGFR (estimated by cystatin C) and the serum creatinine level, suggesting its involvement in renal dysfunction pathways.

### 3.3. Colocalization Analysis and Heidi Test

We conducted colocalization analysis for the two proteins associated with the risk of DKD and found strong evidence of genetic colocalization (PP4 > 0.8), suggesting a high probability for a shared causal variant between protein level and DKD risk. Specifically, our results identified rs66675914 as the shared variant between UMOD and DKD risk and rs11864909 as the shared variant between SIRPA and DKD risk. Furthermore, we used the Heidi test to rule out the potential possibility that this causality was due to two chained disequilibrium SNPs associated with the proteins or DKD (Supplementary Table S5).

### 3.4. Cell Type-Specific mRNA Expression of 2 Target Proteins

**Figure 3:**
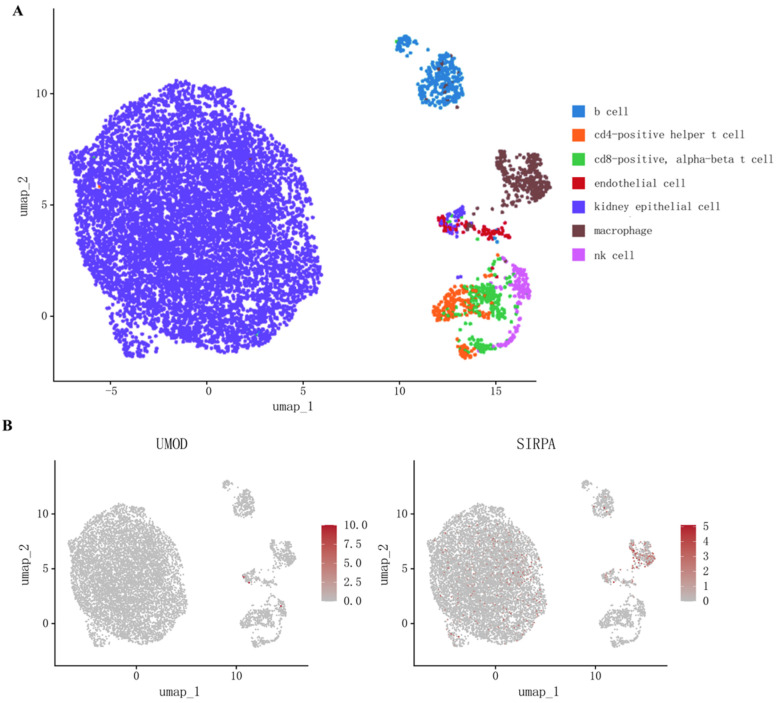
(**A**) Cell types in kidney tissue; (**B**) Single-cell-type expression in kidney tissue for the coding genes of proteins identified by proteome-wide Mendelian randomization.

To explore whether the coding genes of two circulating proteins had any cell type-specific enrichment in the kidney, we further performed single-cell expression analysis using human single-cell RNA-seq data. Our analysis revealed that *SIRPA* was enriched mainly in renal macrophages, whereas the differential expression of *UMOD* was not observed (Figure 3, Supplementary Table S6).

### 3.5. Druggability Evaluation and Association with Current DKD Medications

**Figure 4:**
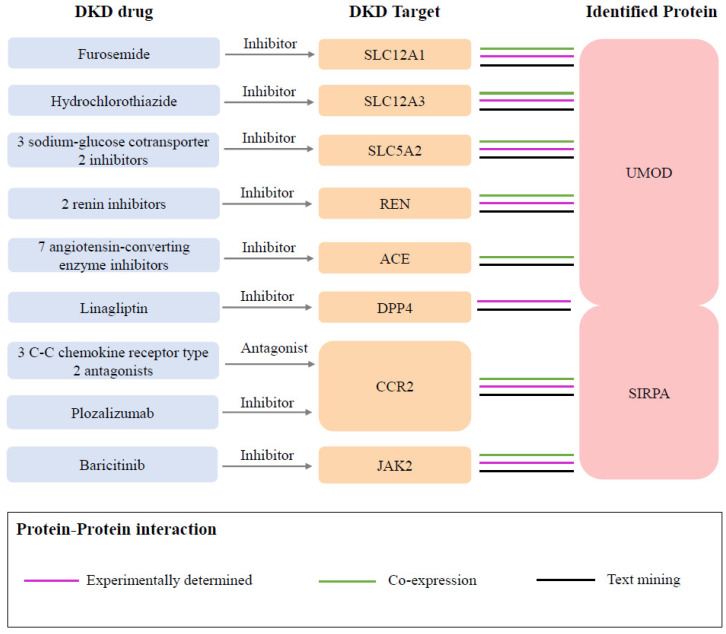
Interactions between current DKD medication targets and identified potential drug targets. Three sodium–glucose cotransporter 2 inhibitors included empagliflozin, canagliflozin, and dapagliflozin. Two renin inhibitors included SPH-3127 and aliskiren. Seven angiotensin-converting enzyme inhibitors included captopril, enalapril, enalapril maleate, fosinopril, lisinopril, perindopril, and ramipril. Three C-C chemokine receptor type 2 antagonists included ilacirnon, PF-04634817, and BMS-813160.

In the druggability evaluation, we found that drugs targeting *SIRPA* have been investigated in clinical trials for the treatment of cancers such as acute myeloid leukemia (evorpacept) and large B-cell lymphoma (maplirpacept) (Supplementary Table S7). We also identified 94 proteins related to known medications indicated for DKD from the Opentarget database (Supplementary Table S8). Through the protein–protein interaction (PPI) network analysis, we found protein interactions between two identified proteins and the targets of nine DKD medications (Figure 4, Supplementary Figure S6). Specifically, *UMOD* was associated with eight target proteins, including *SLC12A1* (targeted by furosemide), SLC12A3 (targeted by hydrochlorothiazide), *SLC5A2* (targeted by three sodium–glucose cotransporter 2 inhibitors), *REN* (targeted by two renin inhibitors), *ACE* (targeted by two renin inhibitors), and *DPP4* (targeted by linagliptin). *SIRPA* was associated with three proteins, including *DPP4*, *CCR2* (targeted by three C-C chemokine receptor type 2 antagonists and plozalizumab), and *JAK2* (targeted by baricitinib).

## 4. Discussion

In this study, we measured 365 circulating cardiometabolic proteins in a Chinese cohort of patients with type 2 diabetes and integrated proteomic and genomic data to investigate the associations between genetically predicted protein levels and DKD risk through MR. We found that genetically high levels of UMOD and SIRPA were associated with an increased risk of DKD, with UMOD replicated in UKB-PPP. Bayesian colocalization results highlighted the causal effects of these proteins. We further verified the differential expression of these protein-coding genes in kidney tissues and found that *UMOD* was enriched predominantly in renal macrophages. Among these two proteins, there was no evidence of drug development for UMOD, whereas SIRPA was associated with drug development for DKD.

The *UMOD* gene encodes uromodulin, a kidney-specific protein, and the most abundant urinary protein. It regulates sodium handling and innate immunity [25,26]. Its levels correlate with nephron mass and eGFR markers [27,28]. In observational studies, higher UMOD values were associated with a lower risk of eGFR decline and incident chronic kidney disease [29,30]. However, GWAS studies link high *UMOD* expression with CKD risk [31,32,33]. Hence, the observational relationship between UMOD levels and kidney function could be biased by reverse causation, and the reduced UMOD levels might be a consequence of decreased kidney function [34]. Recently, one MR study showed that higher UMOD levels are associated with an increased risk of CKD in the general population [35]. We extended this to DKD using a proteome-wide MR approach. Transgenic mice overexpressing *UMOD* develop kidney damage and microalbuminuria. Similar focal lesions have been observed in elderly carriers of *UMOD* risk variants, supporting their role in kidney damage [36].

Another protein identified in the present study was SIRPA, which is expressed in inflammatory cells such as macrophages and dendritic cells [37]. Its overexpression impairs insulin signaling and promotes insulin resistance [38]. SIRPα-CD47 interactions promote inflammation and oxidative stress [39,40]. Since insulin resistance and chronic inflammation are considered to be potential mechanisms underlying DKD [41,42], our findings suggest that SIRPA could have a detrimental effect on DKD. Genetically elevated SIRPA levels are linked to increased DKD risk. Notably, *SIRPA*-targeting drugs such as evorpacept and maplirpacept have been investigated in clinical trials for hematological malignancies. Given the potential role of *SIRPA* in insulin resistance and inflammation, these existing therapies warrant further exploration for their applicability in DKD treatment.

Previously, Zhang et al. investigated 4907 plasma proteins using Somascan-based pQTLs in the Icelanders cohort and identified 21 blood proteins associated with DKD, including three cardiometabolic proteins (C2, TGFBI, and ITIH3) [43]. We did not replicate these findings, likely because of differences in pQTL selection and population heterogeneity.

This study has several strengths, including the proteome-wide MR design based on cis-pQTLs, validation in individuals of European ancestry, and a supportive analysis of colocalization, which provides a better and etiologically important role of associated proteins in disease. However, several limitations should also be considered. First, our study had a small sample size, and additional metabolic proteins associated with DKD might not have been detected. Second, the observed associations might be affected by horizontal pleiotropy, although cis-MR analysis can minimize this bias. Third, the primary analysis was restricted to Asian populations, and the generalizability of our findings to other ancestries warrants further validation.

While our study provides novel insights into the causal role of circulating cardiometabolic proteins in DKD, several future directions should be explored to address its limitations and facilitate clinical implementation. First, larger and multi-ancestry cohorts are needed to validate our findings and assess their generalizability across diverse populations. Second, functional studies using cellular and animal models are crucial to elucidate the precise molecular mechanisms by which UMOD and SIRPA contribute to DKD pathogenesis, which may help identify novel therapeutic targets. Third, given that *SIRPA* has been investigated in oncology for its immunomodulatory role, further research is needed to evaluate its potential as a therapeutic target in DKD. Additionally, drug screening efforts could explore the repurposing of existing SIRPA inhibitors or the development of novel *UMOD*-targeting therapies. Finally, the integration of multiomics approaches, including transcriptomics and metabolomics, may enhance our understanding of the systemic and tissue-specific pathways underlying DKD. These advancements could ultimately lead to the development of precise medicinal strategies for DKD prevention and treatment.

## 5. Conclusions

In conclusion, by integrating proteomic and genomic data, we found that genetically elevated UMOD and SIRPA may be associated with an increased risk of DKD. These findings offer preliminary insights into the etiology of DKD and highlight potential candidates for future research on screening biomarkers and therapeutic targets. Further experimental and large-scale clinical studies are essential to validate the utility and efficacy of these identified proteins.

## Figures and Tables

**Table 1:** Baseline characteristics of patients with type 2 diabetes stratified by DKD.

Characteristics	Overall (*n* = 491)	DKD (*n* = 99)	No DKD (*n* = 392)	*p* Value
Age, y	66.05 (6.89)	65.42 (6.97)	66.21 (6.87)	0.24
Male	257 (52.3)	61 (61.6)	196 (50.0)	0.051
Education (high school)	277 (56.4)	48 (48.5)	229 (58.4)	0.095
BMI, kg/m^2^	25.10 (3.54)	25.61 (3.69)	24.97 (3.49)	0.085
Hypertension	380 (77.4)	86 (86.9)	294 (75.0)	**0.017**
Dyslipidemia	309 (62.9)	65 (65.7)	244 (62.2)	0.61
Current smoking	91 (18.5)	23 (23.2)	68 (17.3)	0.23
Current drinking	91 (18.5)	22 (22.2)	69 (17.6)	0.36
Diabetes duration, y	9.51 (7.11)	10.49 (7.70)	9.26 (6.94)	0.15
HbA1c, mmol/mol	7.43 (1.27)	7.69 (1.42)	7.37 (1.22)	**0.045**
Medication for BP, cholesterol, or diabetes				
Antihypertension	249 (50.7)	56 (56.6)	193 (49.2)	0.23
Cholesterol-lowering ability	96 (19.6)	14 (14.1)	82 (20.9)	0.17
Antidiabetes	332 (67.6)	63 (63.6)	269 (68.6)	0.41

Data are summarized as the means (standard deviations, SDs) for continuous variables or as numbers with proportions for categorical variables. The bold formatting is used to highlight statistically significant results (*p* < 0.05). BMI, body mass index; HbA1c, hemoglobin A1C.

## Data Availability

The datasets used during and/or analyzed during the current study are available from the corresponding author upon reasonable request.

## Supplementary Materials

The following supporting information can be downloaded at https://www.mdpi.com/article/10.3390/biomedicines13040971/s1, Figure S1. A histogram showing the distribution of the range of NPX values, defined as the 90th percentile—the 10th percentile, per protein; Figure S2: Principal component (PC) analysis of Olink Explore data in samples included in the present study. Each dot represents an individual sample; Figure S3: Manhattan plot for genetic associations with plasma UMOD, where the red horizontal line indicates the statistical significance threshold (5 × 10^−8^); Figure S4: Relationship between effect allele frequency (MAF), pQTL effect size, and proportion of variance explained (2*EAF*(1-EAF)*Effect^2^) for 66 independent pQTLs; Figure S5: (A) The effect of tissue-specific protein-coding gene expression on DKD risk for the identified proteins; (B) the association of identified proteins with extensive DKD-related phenotypes; Figure S6: (A) Protein–protein interaction network (PPI) and (B) gene ontology (GO) enrichment pathways among the identified proteins and (C) known DKD medications targets. Tables are available at https://doi.org/10.6084/m9.figshare.25348321.v3 (accessed on 13 April 2025).
