# Supplementary material for: The Identification of Biomarkers and Therapeutic Targets for Diabetic Kidney Disease by Integrating the Proteome with the Genome
Source: Biomedicines. 2025 Apr 16;13(4):971. doi: 10.3390/biomedicines13040971 (PMC12025092; doi:10.3390/biomedicines13040971)
Supplement: Supplementary file 1 [file biomedicines-13-00971-s001.zip › biomedicines-3530212-SI.pdf]

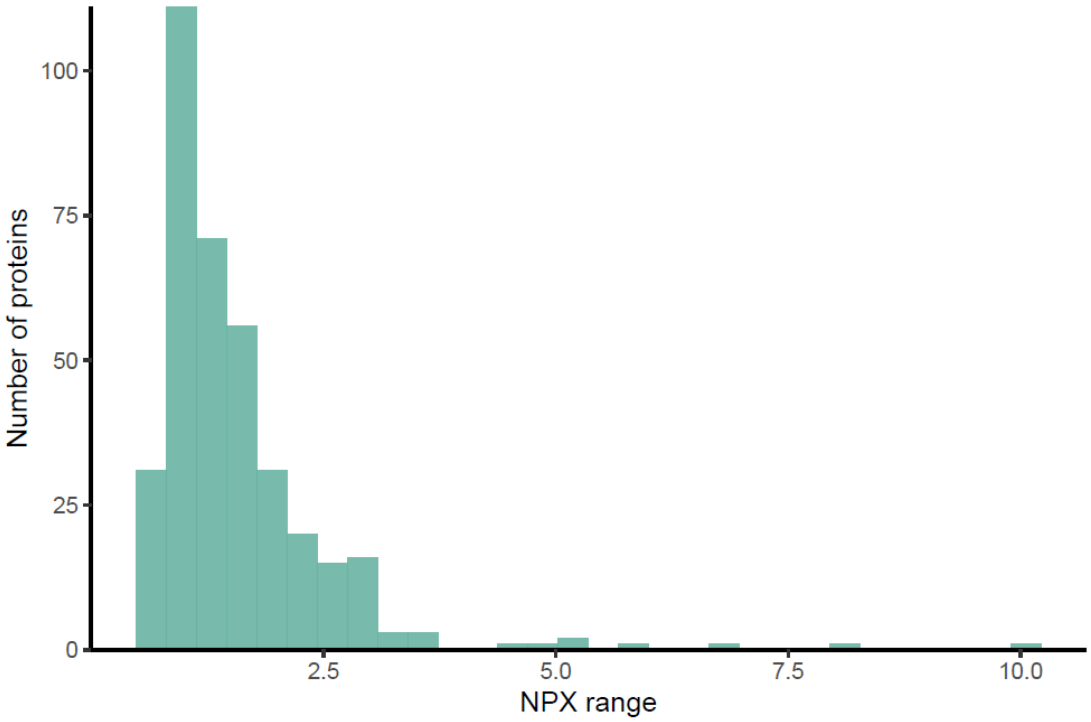

Supplementary Figure S1 The histogram showing the distribution of the range of NPX values, defined as the 90th percentile - the 10th percentile, per protein

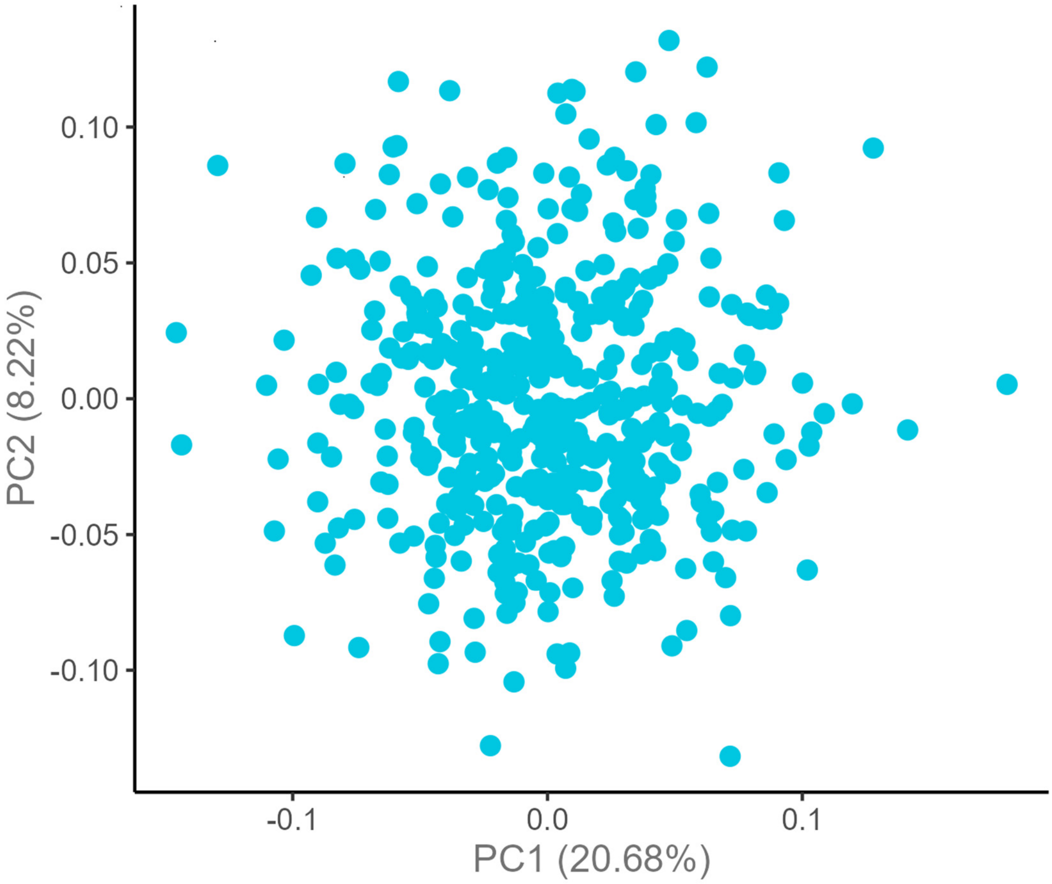

Supplementary Figure S2 Principal component (PC) analysis of Olink Explore data in samples included in the present study

Each dot represents an individual sample

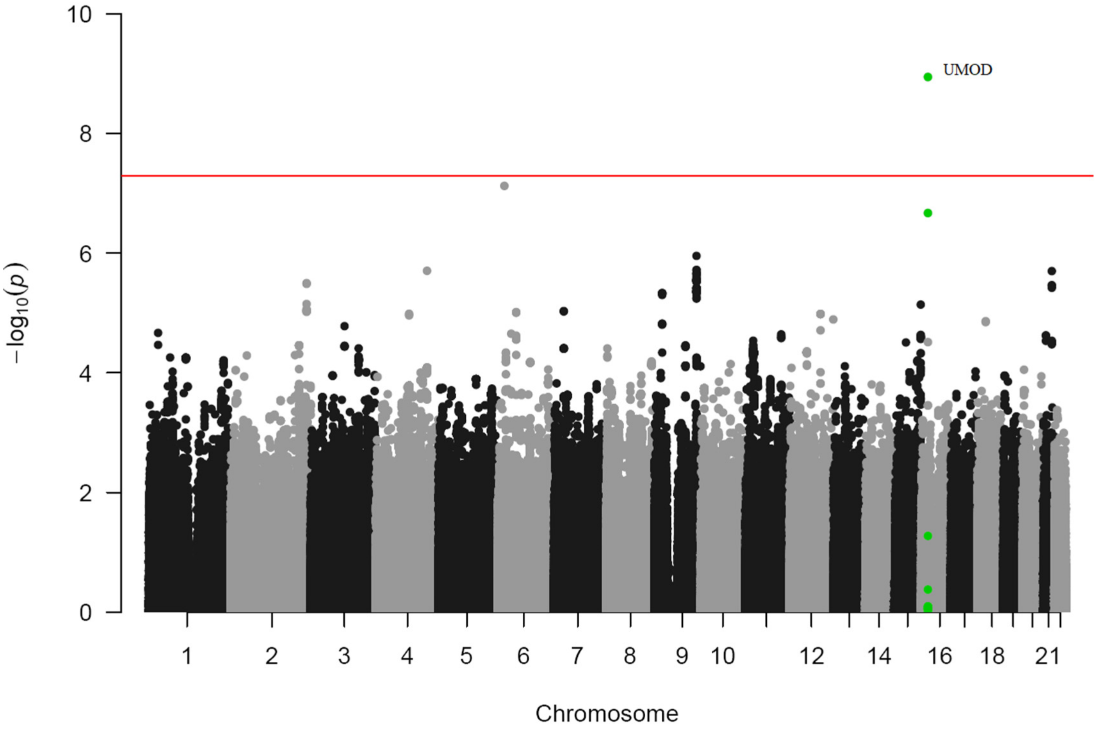

Supplementary Figure S3 Manhattan plot for genetic associations with plasma UMOD, where the red horizontal line indicates the statistical significance threshold (5 × 10^{-8})

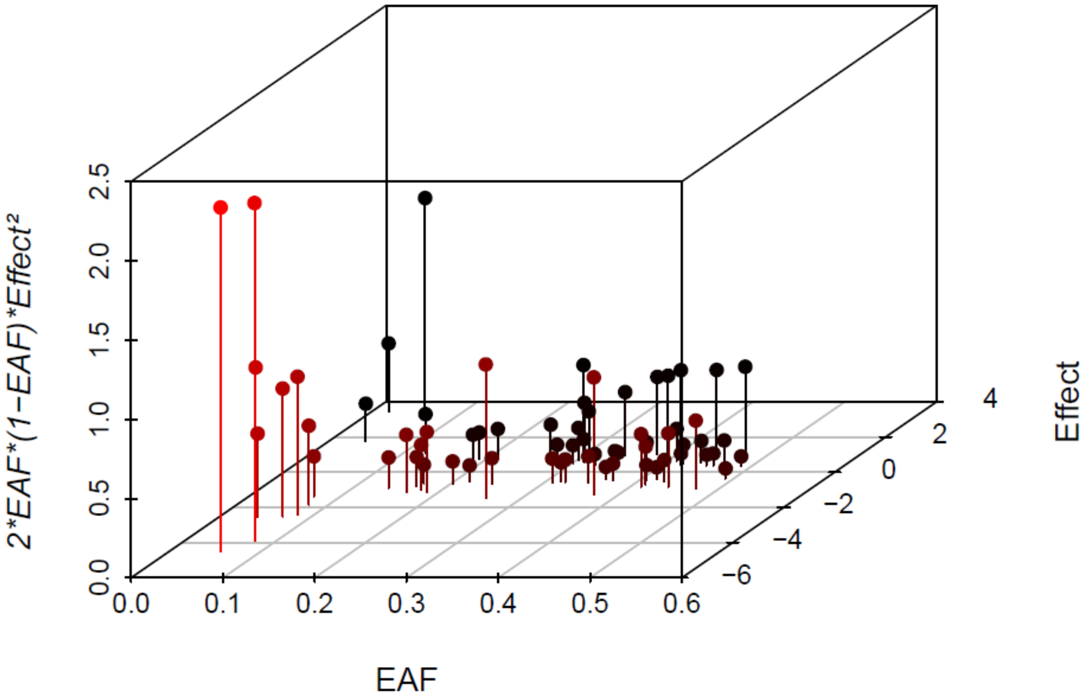

Supplementary Figure S4 Relationship between effect allele frequency (MAF), pQTL effect size and proportion of variance explained (2*EAF*(1-EAF)*Effect^2), for 66 independent pQTLs

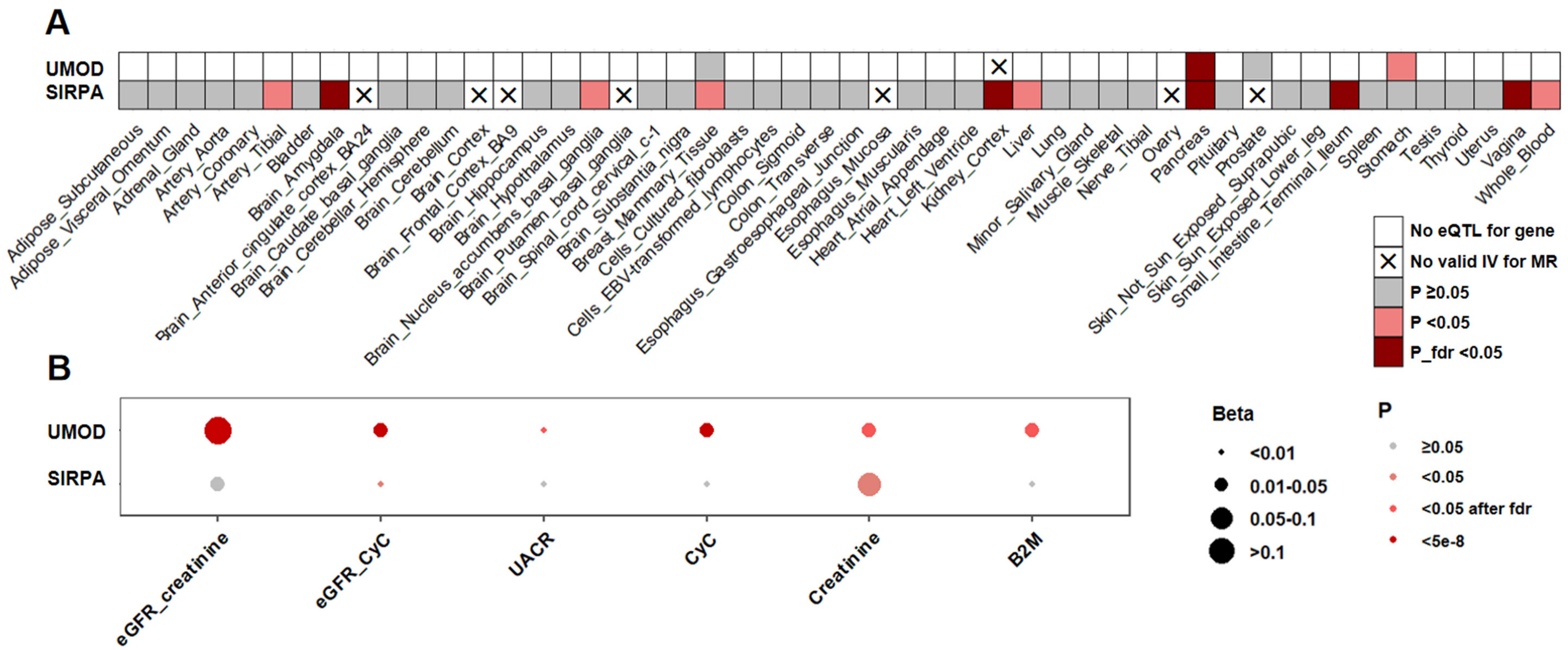

Supplementary Figure S5 A) the effect of tissue-specific protein-coding gene expression on DKD risk for the identified proteins, B) the association of identified proteins with extensive DKD-related phenotypes.

A

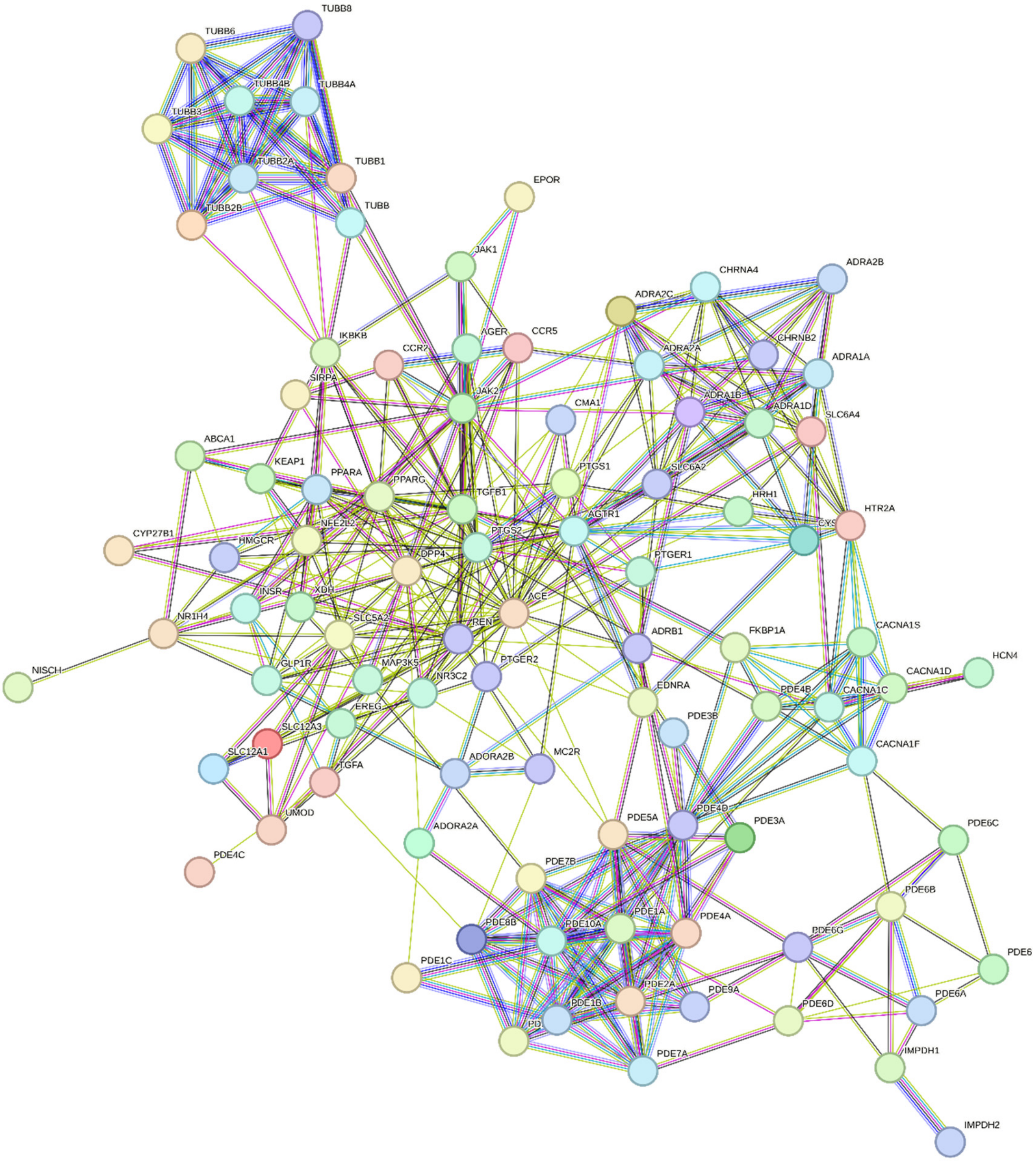
TUBB8
TUBB6
TUBB4B
TUBB4A
TUBB3
TUBB2A
TUBB1
TUBB2B
TUBB
EPOR
JAK1
CHRNA4
ADRA2B
ADRA2C
AGER
CCR5
IKBKB
CCR2
CHRNB2
ADRA2A
ADRA1A
SIRPA
JAK2
CMA1
ADRA1B
ADRA1D
SLC6A4
ABCA1
KEAP1
PPARA
PPARG
TGFB1
PTGS1
SLC6A2
HRH1
HTR2A
CYP27B1
NFE2L2
HMGCR
PTGS2
AGTR1
DPP4
PTGER1
INSR
XDH
ACE
NR1H4
SLC5A2
REN
ADRB1
FKBP1A
CACNA1S
NISCH
GLP1R
MAP3K5
NR3C2
PTGER2
EDNRA
PDE4B
CACNA1D
CACNA1C
HCN4
EREG
PDE3B
SLC12A3
SLC12A1
CACNA1F
TGFA
ADORA2B
MC2R
UMOD
PDE4D
PDE3A
PDE5A
PDE6C
PDE4C
ADORA2A
PDE7B
PDE6B
PDE1A
PDE4A
PDE8B
PDE10A
PDE6G
PDE6
PDE1C
PDE2A
PDE9A
PDE1B
PDE6D
PDE6A
PDE7A
IMPDH1
IMPDH2

B

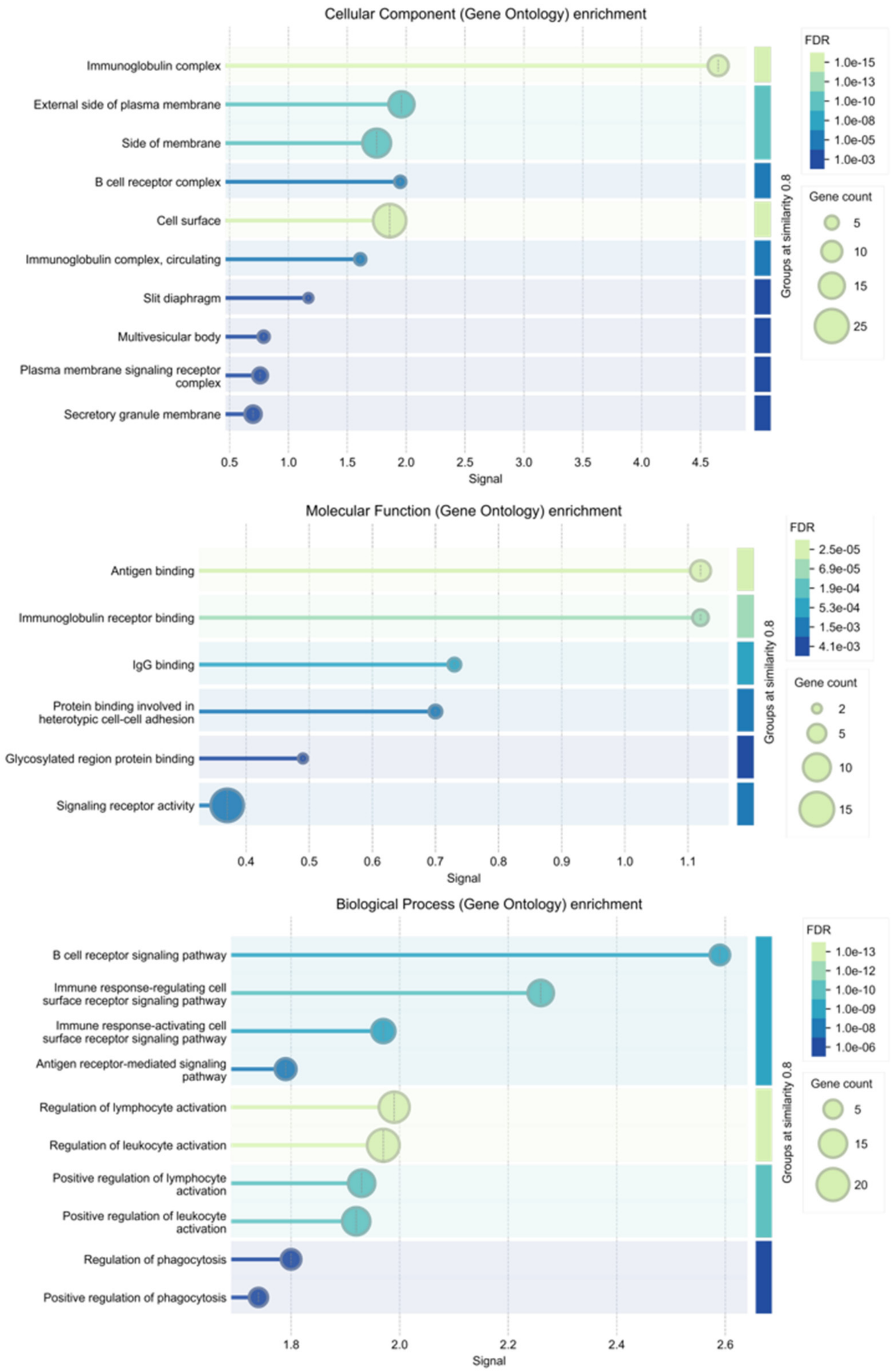

Cellular Component (Gene Ontology) enrichment
Immunoglobulin complex
External side of plasma membrane
Side of membrane
B cell receptor complex
Cell surface
Immunoglobulin complex, circulating
Slit diaphragm
Multivesicular body
Plasma membrane signaling receptor complex
Secretory granule membrane
0.5 1.0 1.5 2.0 2.5 3.0 3.5 4.0 4.5
Signal
Groups at similarity 0.8
FDR
1.0e-15
1.0e-13
1.0e-10
1.0e-08
1.0e-05
1.0e-03
Gene count
5
10
15
25
Molecular Function (Gene Ontology) enrichment
Antigen binding
Immunoglobulin receptor binding
IgG binding
Protein binding involved in heterotypic cell-cell adhesion
Glycosylated region protein binding
Signaling receptor activity
0.4 0.5 0.6 0.7 0.8 0.9 1.0 1.1
Signal
Groups at similarity 0.8
FDR
2.5e-05
6.9e-05
1.9e-04
5.3e-04
1.5e-03
4.1e-03
Gene count
2
5
10
15
Biological Process (Gene Ontology) enrichment
B cell receptor signaling pathway
Immune response-regulating cell surface receptor signaling pathway
Immune response-activating cell surface receptor signaling pathway
Antigen receptor-mediated signaling pathway
Regulation of lymphocyte activation
Regulation of leukocyte activation
Positive regulation of lymphocyte activation
Positive regulation of leukocyte activation
Regulation of phagocytosis
Positive regulation of phagocytosis
1.8 2.0 2.2 2.4 2.6
Signal
Groups at similarity 0.8
FDR
1.0e-13
1.0e-12
1.0e-10
1.0e-09
1.0e-08
1.0e-06
Gene count
5
15
20

C

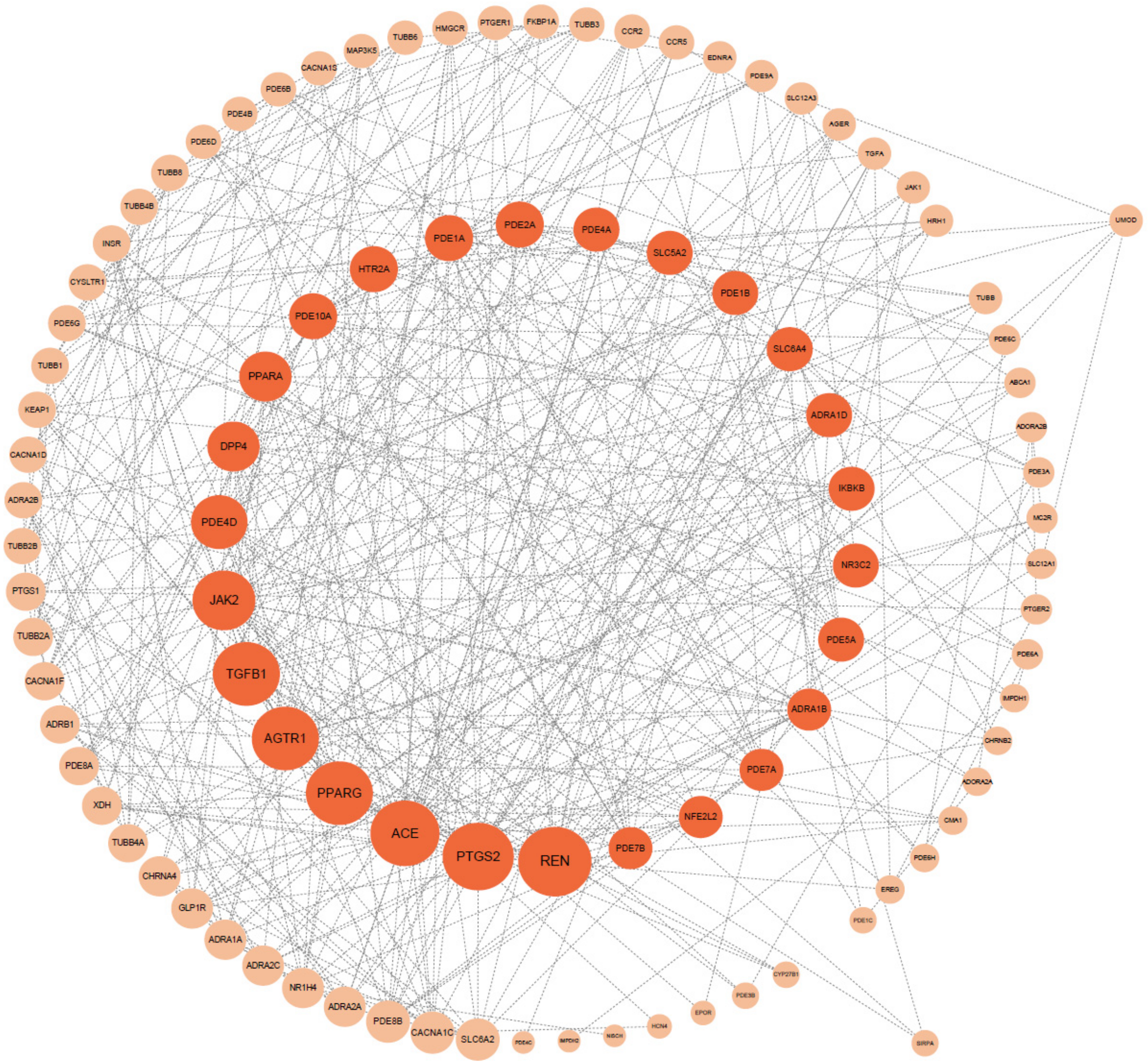

Supplementary Figure S6 A) Protein-Protein interaction network (PPI) and B) Gene Ontology (GO) enrichment pathways among the identified proteins and C) known DKD medications targets
